# The ncBAF Complex Regulates Transcription in AML Through H3K27ac Sensing by BRD9

**DOI:** 10.1158/2767-9764.CRC-23-0382

**Published:** 2024-01-30

**Authors:** David C. Klein, Santana M. Lardo, Sarah J. Hainer

**Affiliations:** 1 Department of Biological Sciences, University of Pittsburgh, Pittsburgh, Pennsylvania.; 2 UPMC Hillman Cancer Center, University of Pittsburgh, Pittsburgh, Pennsylvania.

## Abstract

The non-canonical BAF complex (ncBAF) subunit BRD9 is essential for acute myeloid leukemia (AML) cell viability but has an unclear role in leukemogenesis. Because BRD9 is required for ncBAF complex assembly through its DUF3512 domain, precise bromodomain inhibition is necessary to parse the role of BRD9 as a transcriptional regulator from that of a scaffolding protein. To understand the role of BRD9 bromodomain function in regulating AML, we selected a panel of five AML cell lines with distinct driver mutations, disease classifications, and genomic aberrations and subjected these cells to short-term BRD9 bromodomain inhibition. We examined the bromodomain-dependent growth of these cell lines, identifying a dependency in AML cell lines but not HEK293T cells. To define a mechanism through which BRD9 maintains AML cell survival, we examined nascent transcription, chromatin accessibility, and ncBAF complex binding genome-wide after bromodomain inhibition. We identified extensive regulation of transcription by BRD9 bromodomain activity, including repression of myeloid maturation factors and tumor suppressor genes, while standard AML chemotherapy targets were repressed by inhibition of the BRD9 bromodomain. BRD9 bromodomain activity maintained accessible chromatin at both gene promoters and gene-distal putative enhancer regions, in a manner that qualitatively correlated with enrichment of BRD9 binding. Furthermore, we identified reduced chromatin accessibility at GATA, ETS, and AP-1 motifs and increased chromatin accessibility at SNAIL-, HIC-, and TP53-recognized motifs after BRD9 inhibition. These data suggest a role for BRD9 in regulating AML cell differentiation through modulation of accessibility at hematopoietic transcription factor binding sites.

**Significance:**

The bromodomain-containing protein BRD9 is essential for AML cell viability, but it is unclear whether this requirement is due to the protein's role as an epigenetic reader. We inhibited this activity and identified altered gene-distal chromatin regulation and transcription consistent with a more mature myeloid cell state.

## Introduction

Hematopoietic and myeloid stem cell pools are necessary to populate the circulatory and immune systems; overabundance of these cells, however, can cause a number of myeloproliferative disorders (1–7). Imbalance of hematopoietic differentiation is often rooted in mutations acquired in so-called “leukemic stem cells” that confer proliferative advantages through altered gene expression. Acute myeloid leukemia (AML) is a highly variable disease that results in an overabundance of immature or abnormal blood cells (termed blasts) at the expense of terminally differentiated cells. This imbalanced pool of blood cells occurs as part of a “differentiation block,” which is the main unifying characteristic between distinct AML subtypes (though these subtypes have distinct mechanisms of impaired differentiation; refs. 8, 9). As AML fundamentally represents a failure to complete the myeloid maturation process, the factors regulating leukemogenesis and hematopoietic lineage specification highly overlap, including the MYC, RUNX1, PU.1, C/EBPA, and GATA-family factors (10–14). AML arises through many distinct mechanisms; most AML mutations occur in <10% of patients with AML, and none are known to occur in more than approximately one-third of patients (1, 15). Despite these variable genomic landscapes, key oncogenes are often expressed similarly across patients with AML. For example, overexpression of the *Myc* oncogene is common to over 90% of AML blasts, yet the rate of coding *Myc* mutation in AML cases is only approximately 3% (5, 16–18). It is therefore crucial to understand the factors that regulate oncogene expression beyond unique driver mutations themselves—many of which work through altering the genomic context in which these oncogenes are found.

This genomic context is directly regulated by chromatin-interacting factors, including DNA repair proteins, transcription factors (TF), and nucleosome remodelers. Nucleosome remodelers alter positioning and occupancy of histone proteins to promote or restrict DNA accessibility. Chromatin compaction directly influences gene expression by altering TF binding, access to DNA by RNA polymerase II, and three-dimensional contact between *cis*-regulatory regions (e.g., enhancers and promoters; refs. 19–42). The nucleosome remodeler Brahma-associated factors (BAF or mSWI/SNF) has been implicated in numerous diseases and disorders stemming from improper chromatin structure (43, 44). BAF refers to a family of nucleosome remodeling complexes containing either of two mutually exclusive ATPases, BRG1 or BRM, and many complex subunit variants fulfilling distinct roles (43–50). BAF regulates gene expression, pluripotency, tumor suppression, non-canonical nucleosome structures, and development of the neural, cardiac, and hematopoietic systems (41, 44, 51–57). BAF complex members have been implicated in over 20% of all human cancers, with known interactions with the *Runx1, Myc, Max, Kras,* and *Ras* oncogenes (51, 58). In blood, BAF regulates myeloid and lymphoid differentiation, among other processes (53, 57, 59, 60); BAF mutations alone can deregulate myeloid and lymphoid maturation at numerous steps during differentiation (5, 44, 51, 61–64). BAF complex ATPase inhibitors (BRM011, BRM014, and BRM017) have shown promise for AML therapy, including induced differentiation of independent leukemic cell populations, but with notable off-target effects—likely due to the essential role of BAF in typical hematopoiesis (8, 65). To move toward use of BAF complex inhibitors for cancer therapy, it is necessary to first understand the distinct BAF complex requirements of cancerous and noncancerous myeloid cells.

Recently, one variant of the BAF complex, non-canonical BAF (ncBAF; also known as GBAF) has been identified and shown to regulate the positioning and occupancy of nucleosomes at *cis*-regulatory regions (66–69). ncBAF is distinct from other BAF complexes, as it lacks an AT-rich interaction domain (ARID) subunit that assists in targeting BAF complexes to specific regions of chromatin (36); however, ncBAF uniquely features the bromodomain-containing protein BRD9 (45, 67, 68). Bromodomains recognize acetylated histone residues, which generally loosen chromatin compaction and are associated with activation signatures (70–72). One such posttranslational modification is acetylation of histone H3 at lysine 27 (H3K27ac) which marks active enhancers in cells. Therefore, ncBAF may mediate nucleosome remodeling at H3K27ac-marked regions through enhancer sensing by BRD9. Indeed, the ncBAF complex displays both increased binding to and remodeling activity at H3K27ac-marked peptides when compared with other BAF complex variants (73). Knockdown of BRD9 causes genome-wide dissociation of ncBAF from chromatin (66), but it is unclear how this affects gene transcription, or whether ncBAF is important for enhancer activity. Furthermore, BRD9 is loaded onto the ncBAF complex as a component of the ATPase module, with a crucial scaffolding role for its DUF3512 domain (48, 67). Because of this assembly role for BRD9, depletion of the BRD9 protein does not allow specific interrogation of BRD9-dependent functions, but rather abrogates all ATPase-dependent functions of the ncBAF complex.

To determine BRD9-dependent functions in AML cells, we inhibited acetyl-lysine sensing by the BRD9 bromodomain through the competitive inhibitor I-BRD9 (74), followed by growth assays and genomic assays for BAF complex binding, nascent transcription, and chromatin accessibility. We find that the BRD9 bromodomain activity is required to sustain AML but not HEK293T cell growth. We identified a BRD9 bromodomain activity–dependent portion of the transcriptome, comprising between 5% and 18% of expressed genes, depending on the cell line. This regulation is likely carried out through maintenance of open chromatin at BRD9-bound promoters and gene-distal enhancers, which is disrupted upon BRD9 inhibition. We observe little change to BAF complex binding on chromatin genome-wide after inhibition, with a few notable exceptions. For example, we find that both BRD9 and BRG1 occupancies are reduced at AML-specific enhancers of *Myc,* accompanied by a strong reduction in chromatin accessibility and enhancer RNAs (eRNA) transcription at the individual enhancer elements. We analyzed the differences in chromatin accessibility according to DNA sequence motifs, identifying reduction of accessibility at GATA, ETS, and AP-1 motifs, while SNAIL- and TP53-related motifs are more accessible after BRD9 inhibition. Together, these data suggest that disruption of ncBAF-mediated enhancer architecture alters the regulatory dynamic between enhancers, promoters, and hematopoietic TFs, likely contributing to the defining impairment of differentiation in AML.

## Materials and Methods

### Cell Culture and Media

AML cell lines are all spontaneously immortalized from patient tumor samples, purchased directly from the ATCC (Kasumi-1, KG-1, MV-4-11, and U937) or Sigma (ML-1; refs. 75–79). All authentication of purchased cell lines was performed by the respective suppliers, and no further authentication was conducted. All cell lines were grown in a 5% CO_2_ incubator with 100 rpm shaking in RPMI-1640 (Gibco) containing 2 mmol/L l-glutamine (Gibco), 1 mmol/L sodium pyruvate (Gibco), and 10% FBS (Sigma, 18N103), to a maximum of 40 passages. Stock cultures of cells were stored using a slow-freezing process in typical medium, with 10% DMSO (Thermo Fisher Scientific) as a cryoprotectant. Routine antimycoplasma cleaning was conducted (LookOut DNA Erase spray, Sigma) and cells were screened for *Mycoplasma* presence using PCR.

HEK293T cells (provided by the Fazzio lab) were cultured on tissue culture-treated 10 cm plates in DMEM (Gibco) containing 10% FBS (Sigma, 18N103) and 2 mmol/L glutamine (Gibco). Cells were subcultured via trypsinization approximately every 48 hours and split at a ratio of 1:8 in fresh medium. HEK293T cells were grown in a 5% CO_2_ incubator without shaking.

### Variant Identification

Genomic DNA (gDNA) was extracted from the Kasumi-1, KG-1, ML-1, and MV-4-11 cell lines using the Purelink gDNA Mini Kit (Thermo Fisher Scientific), per manufacturer's instructions. Extracted gDNA was sent to Genewiz for whole-gene sequencing of a panel of *Arid1a, Arid1b, Bcl7a, Bcl7b, Bcl11a, Bcl11b, Brd9, Smarca2*, and *Smarca4*. Sequencing reads were analyzed according to the Genome Analysis Toolkit (GATK) best practices (Broad Institute) and variant impacts were analyzed using SNPEff (80).

### BRD9 Inhibition

I-BRD9 (Selleck S7835, batch no. 01 or Sigma SML1534, lot no. 0000185169, source 0000171479) was dissolved to a stock concentration of 1 mmol/L in 100% DMSO and added to cell culture medium at a 1:100 dilution, for a final working concentration of 10 µmol/L. For each drug treatment, cells were split in equal amounts from a single flask to two T25 flasks, one containing 10 µmol/L I-BRD9 and one containing an equivalent volume of DMSO (Thermo Fisher Scientific; final concentration 1%) as a vehicle control.

### Growth Assay

A total of 50,000 cells from each cell line (Kasumi-1, KG-1, ML-1, MV-4-11, U937, or HEK293T) were initially seeded on a 96-well plate in medium containing 1% DMSO, 10 µmol/L I-BRD9, 2 nmol/L AraC, or 10 µmol/L I-BRD9, and 2 nmol/L AraC. AML cell lines were seeded on non–tissue culture–treated plates in typical RPMI-1640 medium, while HEK293T cells were seeded on tissue culture–treated 96-well plates in described HEK293T medium. At 24-hour intervals, 15 µL of cells were collected from each well, mixed 1:1 with Trypan Blue viability stain (Gibco) and counted using a TC20 automated cell counter (Bio-Rad). Because HEK293T cells are adherent, individual wells were set up for each timepoint and harvested via trypsin digestion, then counted in the same manner as AML cells. Cell counts were normalized to initial plating by dividing the counted cell number by 50,000 to generate a ratio over plated cells. Statistical analyses (two-way ANOVA) were performed in GraphPad PRISM 10.

### Transient Transcriptome Sequencing

Transient transcriptome sequencing (TT-seq) was performed using a modified method (81–84). A total of 500 mmol/L 4-thiouridine (4sU; Carbosynth, T4509) was dissolved in 100% DMSO (Thermo Fisher Scientific). Cells were incubated in RPMI-1640 medium containing 500 µmol/L 4sU at 37°C and 5% CO_2_ for 10 minutes to label nascent transcripts. RNA was then extracted with TRIzol and fragmented using a Bioruptor Pico for one high power cycle. Thiol-specific biotinylation of 100 µg of RNA was performed with EZ-Link Biotin-HPDP (Pierce 21341), dissolved at a concentration of 1 mg/mL in dimethylformamide (Thermo Fisher Scientific) and biotinylation buffer (100 mmol/L Tris-Cl, pH 7.4, 10 mmol/L Ethylenediaminetetraacetic acid [EDTA]). Biotinylation was carried out for 2 hours in the dark with 1,000 rpm shaking at 37°C. RNA was extracted with chloroform and precipitated using NaCl, glycogen, and isopropanol. Labeled RNA was extracted via a pulldown with streptavidin C1 beads (DynaBeads, Thermo Fisher Scientific). Beads were washed in 1 mL of 0.1N NaOH with 50 mmol/L NaCl, resuspended in binding buffer (10 mmol/L Tris-Cl, pH 7.4, 0.3 mol/L NaCl, 1% Triton X-100) and incubated for 20 minutes at room temperature with rotation to bind labeled RNA to beads. Bound beads were washed twice with high salt wash buffer (5 mmol/L Tris-Cl, pH 7.4, 2 mol/L NaCl, 1% Triton X-100), twice with binding buffer, and once in low salt wash buffer (5 mmol/L Tris-Cl, pH 7.4., 1% Triton X-100). Nascent RNA was recovered from beads using a double elution with fresh 100 mmol/L dithiothreitol at 65°C for 5 minutes with 1,000 rpm shaking. Nascent RNA was then extracted with phenol chloroform-isoamyl alcohol and chloroform, and then isopropanol precipitated.

Strand-specific TT-seq libraries were built using the NEBNext Ultra II Directional Library kit, with the following modifications: 200 ng of fragmented RNA was used as input for ribosomal RNA removal via antisense tiling oligonucleotides and digestion with thermostable RNase H (MCLabs; refs. 85, 86). rRNA-depleted RNA samples were DNase treated with Turbo DNase (Thermo Fisher Scientific) and purified by column (Zymo RNA Clean & Concentrator). RNA was fragmented at 94°C for 5 minutes and subsequently used as input for cDNA synthesis and strand-specific library building according to manufacturer protocol. Libraries were pooled and sequenced via Illumina NextSeq2000 to a sequencing depth of approximately 20 million mapped reads.

### TT-seq Data Analysis

Paired-end fastq files were aligned to the hg38 human genome with STAR (options --outSAMtype SAM --outFilterMismatchNoverReadLmax 0.02 --outFilterMultimapNmax 1). Feature counts were generated for GENCODE annotated hg38 genes (V38) using subread featureCounts (options -s 2 -p -B; ref. 87). No filtering for baseline expression was applied because of the sensitivity of TT-seq in detecting lowly expressed transcripts. To visualize TT-seq data, bigwigs were generated using deepTools with transcripts per million (TPM) read normalization (options -bs 1 --normalizeUsing BPM; ref. 88). Reads were imported to R and downstream analysis was conducted using DESeq2 (89). Differentially expressed transcripts were plotted using EnhancedVolcano (90). Significance was defined as DESeq2 adjusted *P* value < 0.05. Principal component analysis (PCA) was performed in R, with and without batch control for cell line performed using limma (91). This correction was only applied to values for PCA shown in Supplementary Fig. S1B. Gene Ontology analysis was performed using Metascape (92), with a background set of all genes expressed in the combined AML cell line panel (DESeq2 baseMean > 1). TPM dot plots were generated in GraphPad Prism 10 and statistical significance was assessed using two-tailed paired *t* tests, with a significance cutoff of 0.01.

### CUT&RUN

CUT&RUN was performed as described previously (93–97), using recombinant Protein A/Protein G-MNase (pA/G-MNase; ref. 98). H3K27ac CUT&RUNs were performed on cells that had been lightly cross-linked with 0.1% formaldehyde (Thermo Fisher Scientific) for 15 minutes and quenched with 0.5 mol/L glycine prior to nuclear extraction. Crosslinks were reversed by 65°C overnight incubation in 0.1% SDS with Proteinase K. All other CUT&RUNs were performed under native conditions. Briefly, 500,000 nuclei were isolated from cell populations in a hypotonic buffer (20 mmol/L HEPES-KOH, pH 7.9, 10 mmol/L KCl, 0.5 mmol/L spermidine, 0.1% Triton X-100, 20% glycerol, freshly added protease inhibitors) and flash-frozen. Nuclei were thawed on ice and bound to lectin-coated concanavalin A magnetic beads (200 µL bead slurry per reaction; Polysciences). Immobilized nuclei were chelated with blocking buffer (20 mmol/L HEPES, pH 7.5, 150 mmol/L NaCl, 0.5 mmol/L spermidine, 0.1% BSA, 2 mmol/L EDTA, fresh protease inhibitors) and washed in wash buffer (20 mmol/L HEPES, pH 7.5, 150 mmol/L NaCl, 0.5 mmol/L spermidine, 0.1% BSA, fresh protease inhibitors). Nuclei were incubated in wash buffer containing primary antibodies for 1 hour at room temperature with rotation. Primary antibodies targeting BRD9 (Invitrogen PA5-113488, lot WB3192114), BRG1 (Bethyl A300-813A, lot 5), BRM (Cell Signaling Technology 11966S, lot 5), BAF57 (Bethyl A300-810A, lot 2), and H3K27ac (Abcam ab4729, lot GR3357415-3) were used in the amount of 1 µg per sample. Nuclei were washed and incubated in wash buffer containing recombinant pA/G-MNase for 30 minutes at room temperature with rotation to bind to engaged primary antibody. Untargeted controls lacking primary antibody were subjected to the same conditions but incubated in wash buffer without antibody prior to pA/G-MNase incubation. Samples were equilibrated to 0°C and 3 mmol/L CaCl_2_ was added to activate pA/G-MNase cleavage. After digestion for 15 minutes at 0°C, digestion was chelated with 20 mmol/L EDTA and 4 mmol/L egtazic acid (EGTA), and 1.5 pg MNase-digested *S. cerevisiae* mononucleosomes were added as a spike-in control. Genomic fragments were released through either RNase A treatment or salt fractionation with subsequent RNase A treatment. After separating released fragments through centrifugation, fragments isolated were used as input for a library build consisting of end repair and adenylation, NEBNext stem-loop adapter ligation, and subsequent purification with AMPure XP beads (Beckman Coulter). Barcoded fragments were then amplified by 14 cycles of high-fidelity PCR and purified using AMPure XP beads. Libraries were pooled and sequenced on an Illumina NextSeq2000 to a depth of approximately 10 million mapped reads.

### CUT&RUN Data Analysis

CUT&RUN data were analyzed as described previously (93, 94, 96). Paired-end fastq files were trimmed to 25 bp and mapped to the hg38 genome with bowtie2 (options -q -N 1 -X 1000 --very-sensitive-local; ref. 99). Mapped reads were filtered for PCR duplicates via Picard (100) (RRID:SCR_006525) and filtered for MAPQ ≥10 using SAMtools (101). Size classes corresponding to BAF complex footprints (<200 bp) or histones (150–500 bp) were generated using a custom awk script and SAMTools (101). Reads were converted to bigWig files using deepTools with read normalization to 1x coverage (options -bs 5 --smoothLength 20 --normalizeUsing RPGC, --effectiveGenomeSize 2862010578; ref. 88). Heat maps were generated using deepTools computeMatrix (options -a 2000 -b 2000 -bs 20 --missingDataAsZero) and plotHeatmap (88). Peaks were called from CUT&RUN data using SEACR, a CUT&RUN-specific peak-calling algorithm with relaxed stringency and controls lacking primary antibody (analogous to input DNA for a ChIP-seq experiment; ref. 98).

### Analysis of Public Chromatin Immunoprecipitation Sequencing Data

Chromatin immunoprecipitation sequencing (ChIP-seq) data were analyzed as described previously. Publicly available ChIP-seq data were downloaded from the NCBI Sequence Read Archive (SRA), accession number PRJNA751732 (102). Paired-end fastq files were trimmed to 25 bp, while single-end fastq files were not trimmed. All fastq files were mapped to the hg38 genome using bowtie2 (options -q -N 1 -X 1000 --very-sensitive-local; ref. 99). Mapped reads were filtered for MAPQ ≥ 10 using SAMtools (101). Mapped reads passing QC checks were converted to bigWig files using deepTools with read normalization to 1x coverage (options -bs 5 --smoothLength 20 --normalizeUsing RPGC, --effectiveGenomeSize 2862010578; ref. 88). Heat maps were generated using deepTools computeMatrix (options -a 2000 -b 2000 -bs 20 --missingDataAsZero) and plotHeatmap (88).

### Assay for Transposase-Accessible Chromatin using Sequencing

Omni-ATAC-seq was performed as described previously, with modification (103). A total of 60,000 nuclei were extracted as described above for CUT&RUN (native) and flash-frozen until use. Frozen nuclei were resuspended in transposition mix containing 1X TD buffer (10 mmol/L Tris pH 7.6, 5 mmol/L MgCl_2_, 10% dimethylformamide), DPBS, 0.1% Tween-20, 1% digitonin, and 4 µL Tn5 transposome (Diagenode) per reaction. Samples were incubated at 37°C for 30 minutes with 1,000 rpm shaking. Transposed DNA was purified using a Clean and Concentrator kit (Zymo) per manufacturer's instructions. Samples were amplified for five cycles of high-fidelity PCR (KAPA), then held on ice and assessed via qPCR (KAPA SYBR Green). Samples were then returned to the thermocycler for as many cycles as needed to reach 1/3 qPCR saturation (∼10 total cycles). Amplified libraries were gel-extracted between 150 and 650 bp and sequenced via Illumina NextSeq2000 to a depth of approximately 50 million mapped reads.

### Assay for Transposase-Accessible Chromatin using Sequencing Data Analysis

Paired-end fastq files were analyzed Assay for Transposase-Accessible Chromatin using sequencing (ATAC-seq) data using PEPATAC, via the standard analysis pipeline (104). PEPATAC was used to quality-check the ATAC-seq datasets, ensuring that all replicates had transcription start site (TSS) enrichment scores of >10. We then converted reads to bigwig files using deepTools bamCoverage (options --normalizeUsing RPGC, --effectiveGenomeSize 2833823455, --binSize 1 --smoothLength 4, --centerReads -e) from the sorted, deduplicated BAM file generated by PEPATAC (88, 104). Heat maps were generated using deepTools computeMatrix (options -a 2000 -b 2000 -bs 20 --missingDataAsZero) and plotHeatmap (88). To identify altered TF footprints among ATAC-seq datasets, data were processed using TOBIAS according to the standard analysis pipeline of ATACorrect, FootprintScores, and BINDetect, using the JASPAR2022 core vertebrate collection of DNA sequence motifs and merged peaks generated by the PEPATAC pipeline (104–106). Deduplicated bam files were merged from all cell lines for this analysis (*n* = 20).

### Materials and data availability

The raw and processed sequencing data generated in this study have been deposited in the NCBI Gene Expression Omnibus under the SuperSeries GSE241428. Publicly available ChIP-seq data analyzed in this study were obtained from the SRA under the accession number PRJNA751732.

No plasmids or cell lines were generated in this study, but materials are available on request. All resources must be acquired via a Material Transfer Agreement granted by the University of Pittsburgh (Pittsburgh, PA).

## Results

### BRD9 Bromodomain Function is Required for AML Cell Growth

BRD9 is overexpressed in AML cells, which display reduced viability upon depletion of BRD9 (4, 66). However, BRD9 plays a dual role in ncBAF complex via the DUF3512 scaffolding domain and histone acetylation recognition bromodomain and therefore it is unclear whether BRD9 scaffolding or recognition activity are required for AML viability. In this study, we set out to determine the role of the BRD9 bromodomain, selecting a panel of five AML cell lines representing various morphologic classes (Table 1) to interpret results based on a range of phenotypes and driver mutations.. To limit the possibility of unknown mutations compromising BAF complex function in the cell lines selected for this panel, we performed whole-gene sequencing of selected relevant genes in the Kasumi-1, KG-1, ML-1, and MV-4-11 cell lines (*Arid1a, Arid1b, Bcl7a, Bcl7b, Bcl11a, Bcl11b, Brd9, Smarca2,* and *Smarca4*; Supplementary Table S1). We assigned variant effect predictions using SNPEff (80) and found only one high-impact variant: the previously identified frame-shift mutation truncating the C-terminal region of the Kasumi-1 BRG1 protein. Approximately 95% of all variants identified were noncoding, and of the 5% that were in coding regions, approximately 80% were predicted to be of low consequence in each cell line.

**TABLE 1:** AML characteristics of selected cell line panel

Cell line	Kasumi-1	KG-1	ML-1	MV-4-11	U937
FAB classification	M2	M6	M4	M5	M5
Driver mutation	*Runx1-Eto*	*Fgfr1op2-Fgfr1* fusion	*Mll-Af6* fusion	*Mll-Af4* fusion	*Calm-Af10* fusion

NOTE: FAB = French American British system for AML status classification indicating the type of cell from which the leukemia originated and the extent of leukemic cell maturation.

**FIGURE 1:**
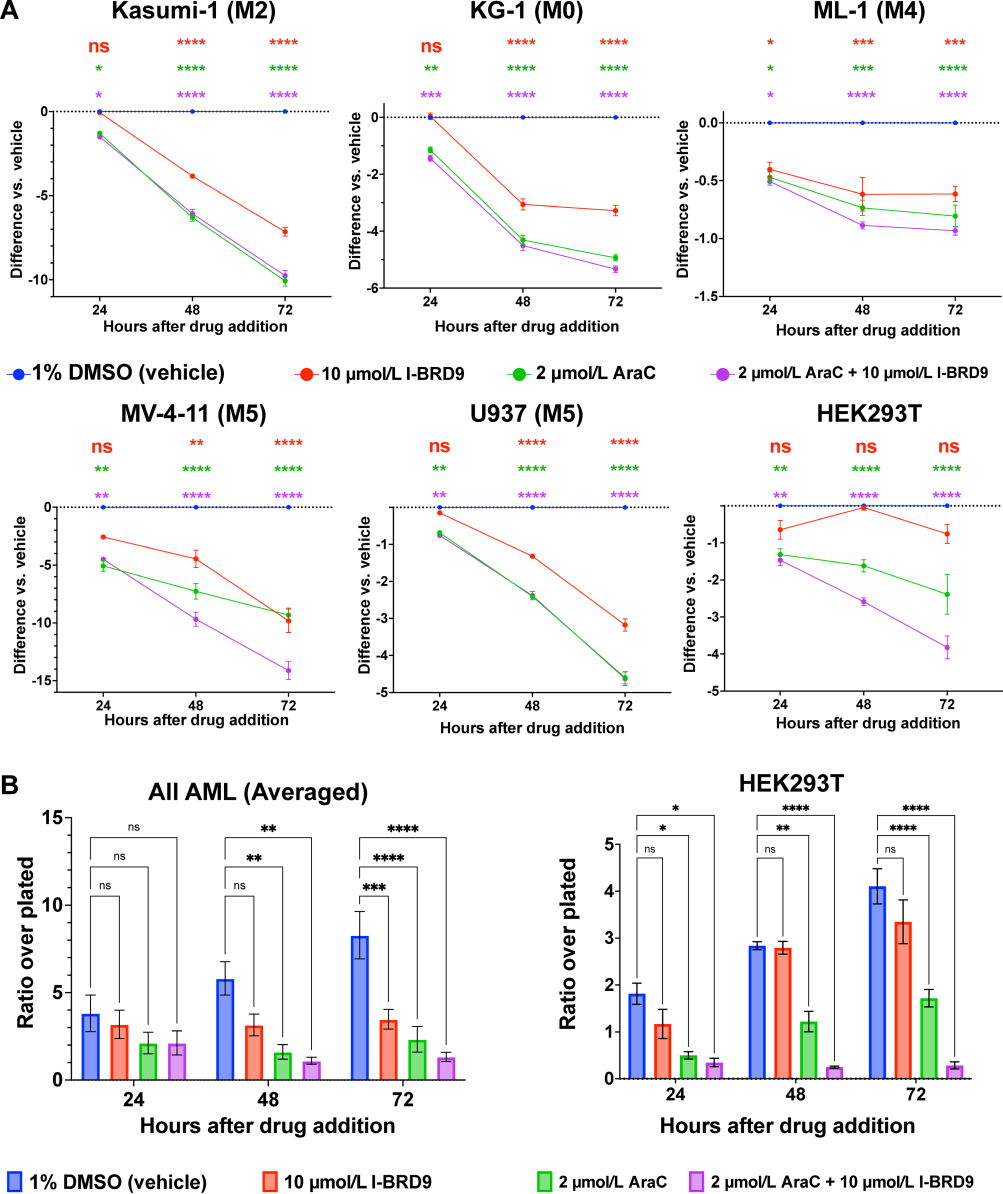
Inhibition of the BRD9 bromodomain selectively kills AML cells but not HEK293T cells. **A,** Growth assays depicting difference in ratio of cells remaining after indicated hours of growth in medium containing 1% DMSO (vehicle), 10 µmol/L I-BRD9, 2 µmol/L cytarabine (AraC), or a combination. *n* = 4 replicates per drug condition and cell line (HEK293T, *n* = 3). *Y*-axis indicates ratio of cells counted to those originally plated, compared with vehicle. Points are shown ± SEM. **B,** Averaged results of growth assays across all cell lines (left) and HEK293T cells (right). Error bars indicate SEM. (*n* = 20 per drug condition, analyzed via two-way ANOVA. *, *P* < 0.05; **, *P* < 0.01; ***, *P* < 0.001; ****, *P* < 0.0001; ns, *P* > 0.05).

To determine whether BRD9 is important for AML viability through the action of the bromodomain, we performed growth assays over a time course of treatment with I-BRD9, a competitive inhibitor specific to the bromodomain of BRD9 (74). We find that BRD9 bromodomain inhibition effectively reduces cell growth in all five AML cell lines, paralleling viability defects observed in BRD9 depletion (4, 66, 107) with much milder effects in HEK293T cells (Fig. 1A). These data suggest that BRD9 regulates AML viability through its bromodomain, rather than solely as a scaffold for ncBAF complex assembly. We compared these effects on cell growth with those resulting from treatment with the approved AML chemotherapy drug cytarabine (AraC). AraC treatment resulted in a greater reduction in cell growth than I-BRD9; however, AraC was also more potent than I-BRD9 in HEK293T cells, as expected on the basis of the non–AML-specific mechanism of AraC action (cytidine analog incorporation; ref. 108). We combined treatment with I-BRD9 and AraC to determine whether growth inhibition is additive or synergistic; however, combination treatment rarely produced stronger effects than AraC treatment alone in AML cell lines. Surprisingly, there was an additive effect on HEK293T cell growth upon addition of both AraC and I-BRD9; we suspect this to be due to a role for BRD9 in DNA repair, which has been identified through rare melanoma variant screening (109). To quantify the general effects of BRD9 bromodomain inhibition on AML cells, we averaged the results presented in Fig. 1A across all AML cell lines and compared these results with the effects on HEK293T cells (Fig. 1B). In agreement with prior work examining *Brd9* knockdown (4, 74), we find that BRD9 inhibition significantly reduces AML cell count, while the effects are not significant in HEK293T cells unless AraC is added (Fig. 1B). The efficacy and specificity of BRD9 inhibition on AML cell growth suggest an AML-specific vulnerability that may be valuable as a target for novel chemotherapeutic approaches.

### BRD9 Regulates the AML Transcriptome Through its Bromodomain

We next examined a possible mechanism for BRD9’s role in sustaining AML cell growth and characteristic impairment of differentiation. To avoid indirect effects of BRD9 inhibition, we shortened our inhibitory time course to a minimal effective treatment of 6 hours at 10 µmol/L, validated in Kasumi-1 cells to be above the IC_50_ for I-BRD9 (74). To further focus our approach on direct effects of BRD9 inhibition, we profiled the AML transcriptome using the nascent RNA-sequencing technique TT-seq (81). We validated replicate similarity using PCAs, identifying strong grouping according to cell line (Supplementary Fig. S1A). When cell line–specific (but not treatment-dependent) transcription was batch-corrected using limma (91), samples separated out according to treatment condition, demonstrating a shared shift in transcriptome upon BRD9 bromodomain inhibition (Supplementary Fig. S1A). We analyzed differential transcription among each cell line individually using DESeq2 (Table 2; Supplementary Fig. S1B–S1F; Supplementary Table S2; ref. 89). Intriguingly, the effect of BRD9 inhibition on transcription qualitatively correlates with maturation state; cells with maturation to monocytic/macrophage stages were most affected (the two FAB-M5 lines, MV-4-11 and U937, being most affected), while the relatively undifferentiated cell lines (KG-1 and ML-1) were less sensitive to BRD9 bromodomain inhibition (Table 2). We examined the conservation of differentially transcribed genes across cell lines and found that over 60% of differentially transcribed genes were affected similarly in at least two cell lines upon I-BRD9 treatment (log_2_ fold change in the same direction, adj. *P* < 0.05), suggesting a conserved mechanism of BRD9 function across AML cell lines.

**TABLE 2:** Differentially transcribed genes after 6-hour treatment with 10 µmol/L I-BRD9

Cell line	Kasumi-1	KG-1	ML-1	MV-4-11	U937
BRD9-stimulated	1,380	772	883	2,599	2,993
BRD9-repressed	1,161	716	555	1,340	2,480

NOTE: BRD9-stimulated = positive log_2_ fold change and adj. *P* < 0.05 after I-BRD9 treatment. BRD9-repressed = negative log_2_ fold change and adj. *P* < 0.05 after I-BRD9 treatment. *n* = 2 per condition for each cell line, for a total of 20 replicates. Total: 30,943 expressed genes (baseMean ≥ 1 in batch-analyzed output)

**FIGURE 2:**
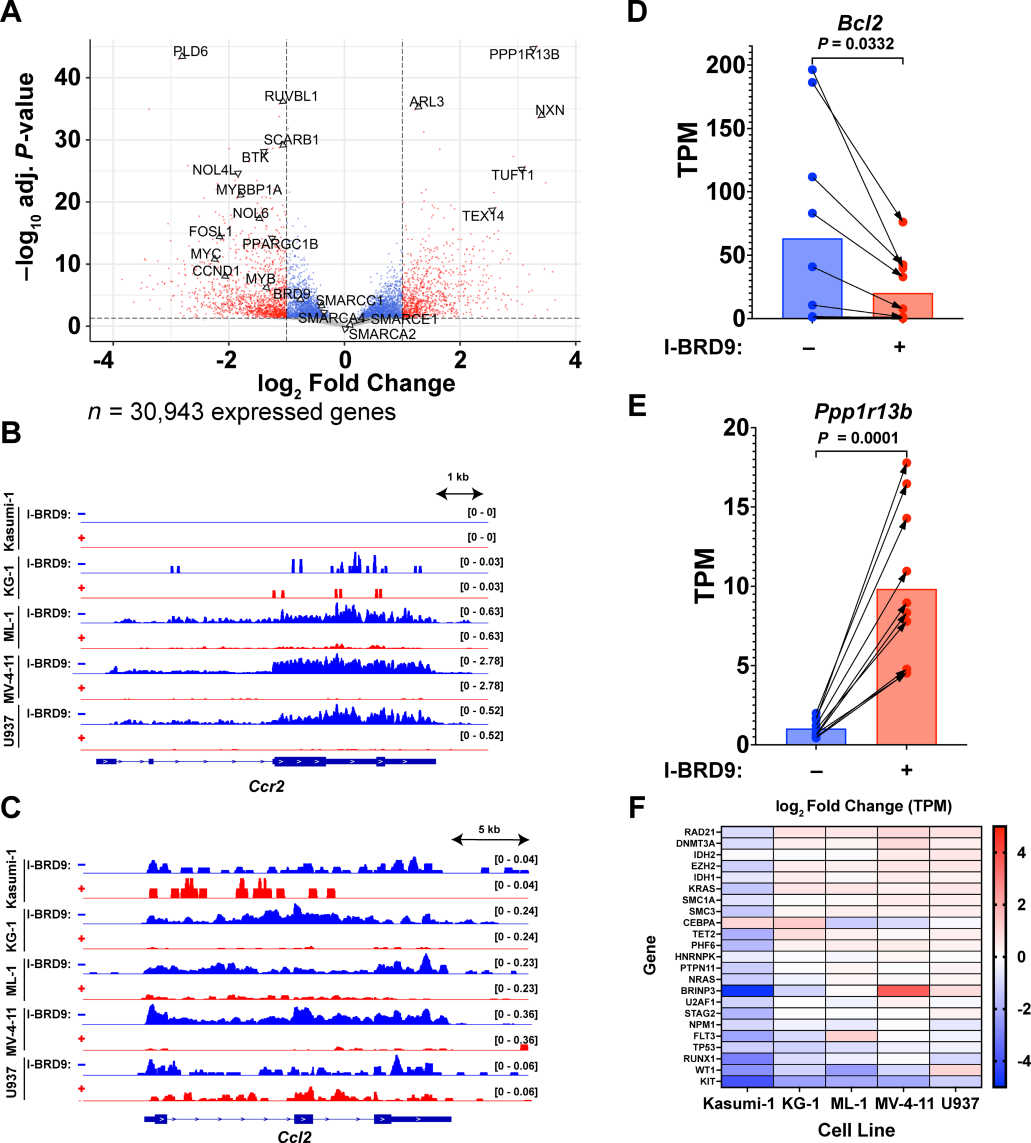
The BRD9 bromodomain regulates transcription of AML- and hematopoiesis-associated genes. **A,** Volcano plot depicting DESeq2 results from TT-seq experiments in Kasumi-1, KG-1, ML-1, MV-4-11, and U937 AML cell lines, analyzed together. Fold changes are shown as effect after 6-hour treatment with 10 µmol/L I-BRD9, relative to vehicle (1% DMSO). Significance cutoffs of adj. *P* < 0.05 and log_2_ fold change > 1 are indicated by dashed lines. *n* = 2 replicates per drug condition and cell line, for a total of 20 experiments. Browser tracks depicting transcription of the *Ccr2* (**B**) and *Ccl2* (**C**) genes. *n* = 2 replicates per condition and cell line, averaged. TPM values of the FDA-approved AML chemotherapy target *Bcl2* (**D**) and p53-related tumor suppressor gene *Ppp1r13b* (**E**). Arrows link the same replicate between conditions. *n* = 2 replicates per drug condition and cell line, for a total of 20 experiments. Significance was assessed using paired two-tailed *t* tests. **F,** Heatmap depicting log_2_ fold change of TPM values after 6-hour I-BRD9 treatment for 23 genes defined as significantly mutated in AML per TCGA guidelines (118). *n* = 2 replicates per condition and cell line.

To identify more general effects of BRD9 inhibition on transcription in AML cells rather than focusing on cell type specific alterations, we combined replicates from the individual cell lines and performed a single-batch analysis of differential transcription using DESeq2. We identified 5,819 differentially transcribed genes, of which 2,800 (9.05% of 30,943 expressed genes) were upregulated by BRD9 inhibition, while 3,019 (9.76% of 30,943 expressed genes) were downregulated (Fig. 2A). In line with previous reports (4, 74), we identified reduced transcription of *Myc* after I-BRD9 treatment, as well as the neighboring long noncoding RNA (lncRNA) *Ccdc26*, transcription of which is suppressed during differentiation of HL60 myeloid leukemia cells (110). Intriguingly, *Ccr2* and *Ccl2*, two proteins that are preferentially expressed in tumor cells and are critical for cancer cell proliferation (111, 112), were strongly downregulated (Fig. 2B and C), suggesting a potential mechanism through which I-BRD9 inhibits AML but not HEK293T cell growth.

To further examine these data, we calculated TPM values for each gene and individual TT-seq replicate (Supplementary Table S2). We selected three genes that are targeted by FDA-approved inhibitors for treatment of leukemia: *Bcl2* (inhibited by Venetoclax), *Btk* (Ibrutinib), and *Kit* (Imatinib) and plotted the calculated TPM values for each TT-seq replicate after DMSO and I-BRD9 treatment, connected by arrows (Fig. 2D; Supplementary Fig. S1G and S1H). For each target, TPM values were significantly reduced by I-BRD9 treatment (*P* = 0.0332, *P* < 0.0001, *P* = 0.0086 for *Bcl2, Btk,* and *Kit*, respectively), suggesting that the BRD9 bromodomain may be a relevant clinical target for drug therapy. Conversely, we examined three tumor suppressor genes: the *Tp53*-related *Ppp1r13b*, the base excision repair polymerase *Polβ*, and the antiproliferative coactivator of cell differentiation *Btg1* (Fig. 2E; Supplementary Fig. S1I and S1J). TPM-normalized transcription of each gene was significantly upregulated by BRD9 inhibition (*P* = 0.0001, *P* = 0.0044, *P* = 0.0043 for *Ppp1r13b*, *Polβ*, and *Btg1*, respectively), indicating possible roles for the BRD9 bromodomain in suppression of DNA repair and cell-cycle checkpoint evasion in AML. BRD9-mediated repression of *Btg1* transcription suggests that BRD9 may have a role in preventing differentiation of AML cells that is reminiscent of ncBAF's role in maintaining pluripotency in embryonic stem cells (68).

To examine biological pathways, using all significantly altered genes, we performed Gene Ontology term analysis, identifying upregulation of pathways associated with autophagy, cell division, and signaling—including by Rho GTPases and mTOR, both implicated in cancer (113–117). Pathways associated with leukocyte activation, translation, and targets of MYC activation were downregulated after BRD9 inhibition, among others (Supplementary Fig. S2A and S2B). Finally, we assessed transcriptional changes for the 23 genes identified as significantly mutated in AML by the Cancer Genome Atlas (TCGA) after BRD9 inhibition (Fig. 2F; ref. 118). Transcription of *Kit, Wt1, Runx1*, *Npm1*, and *Flt3* were reduced in at least four of five cell lines, suggesting a broadly effective manner of chemotherapeutic potential for BRD9 inhibitors. Together, these data suggest that ncBAF is responsible for maintaining the AML transcriptome through the BRD9 bromodomain, a mechanism with potential for selective anticancer cell activity.

### The BRD9 Bromodomain is Necessary to Maintain the Immature States of AML Cells

**FIGURE 3:**
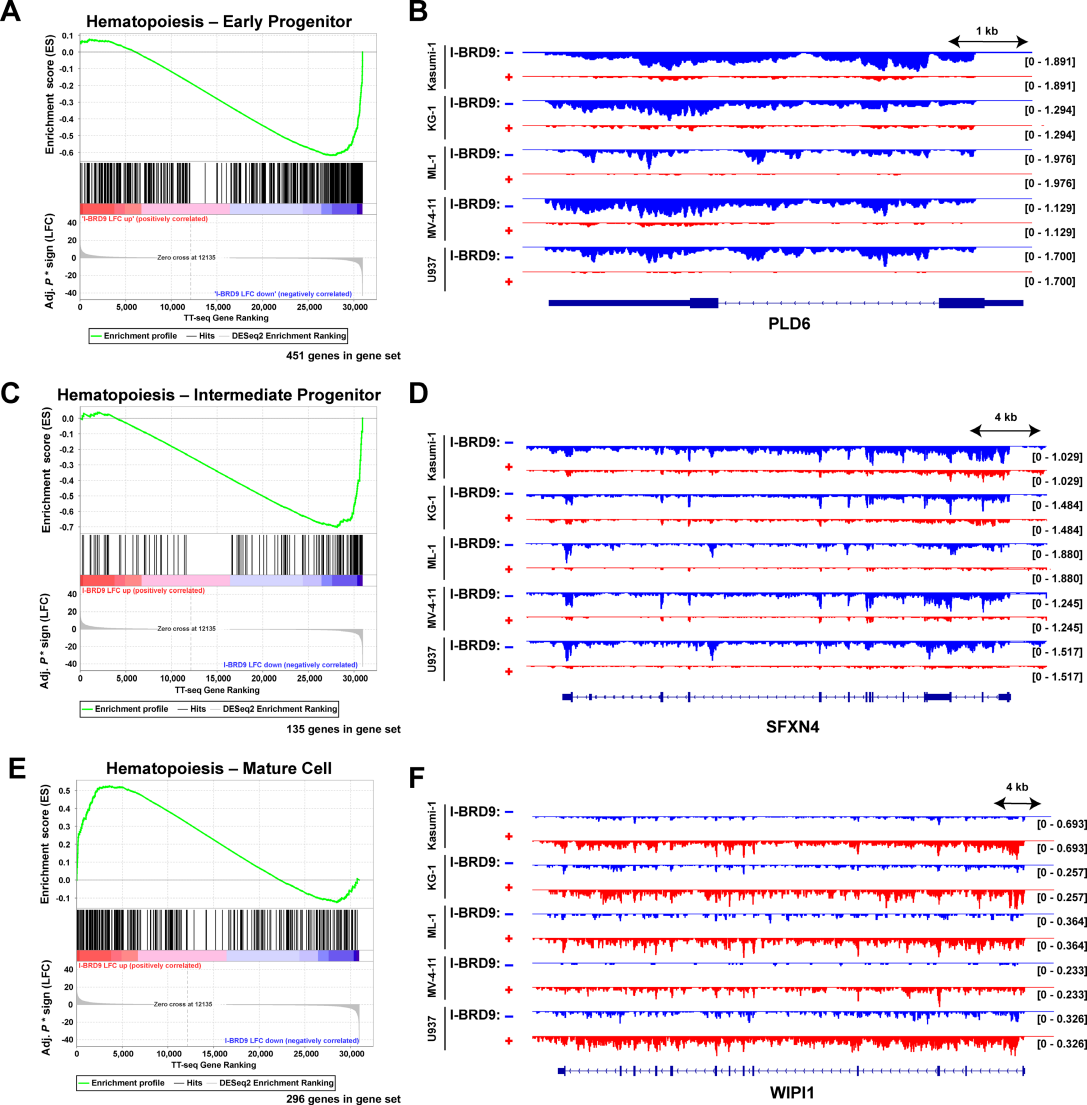
BRD9 maintains immature myeloid gene transcription in AML cells. GSEAs and representative browser tracks assessing hematopoietic cell maturity gene signatures depicting transcription at the associated hematopoietic maturation stage for early progenitor (**A** and **B**), intermediate (**C** and **D**), and mature (**E** and **F**) blood cells. TT-seq gene rankings: (–log_10_ (adj. *P*, * sign of fold change). *n* = 2 replicates per condition and cell line, merged for analysis. To the right are individual browser tracks for one gene in each gene set which depict strand-specific transcription oriented to the indicated gene (all examples are transcribed on the reverse strand).

To determine how BRD9 sustains AML cell viability, we performed gene set enrichment analyses (GSEA) using a list of differentially transcribed genes from all combined cell lines [ranked by −log_10_ (adj. *P*) * sign of fold change]. Among the most significantly enriched gene sets were three hematopoiesis-related gene sets: gene signatures associated with early and intermediate progenitor cells were reduced after 6-hour I-BRD9 treatment, while mature hematopoietic cell gene signatures were increased after I-BRD9 treatment (Fig. 3A, C, and E). The defining characteristic of AML is impaired differentiation preventing full myeloid maturation, with distinct differentiation blocks by AML subtype (2, 3, 119–122). Enrichment of mature myeloid gene signatures therefore suggests that BRD9 inhibition alters not just the AML transcriptome, but the differentiation state of AML cells as well. To validate these trends, we visualized transcription in each cell line at a gene associated with early hematopoietic progenitor cells (*Pld6*, Fig. 3B), intermediate progenitor cells (*Sfxn4*, Fig. 3D), and mature blood cells (*Wipi1*, Fig. 3F), confirming that early and intermediate progenitor gene transcription is reduced in all cell lines, while mature blood cell transcription is increased in all cell lines. We interpret these results as suggesting a role for BRD9 in maintenance of AML cells in their state of incomplete differentiation, a role which may explain the specific requirement for BRD9 in AML cells.

### BRD9 Bromodomain Activity Regulates Genes Through Maintenance of Accessibility at Distal Enhancers and Promoters

We hypothesized that the characteristic AML differentiation block may be potentiated through gene-distal regulation, as gene-distal elements, such as enhancers, display well-established dysregulation in many cancer types, including blood cancers (5, 8, 123–126). More specifically, we hypothesized that enhancers regulating blood differentiation are bound by ncBAF, an interaction likely regulated through recognition of H3K27ac by the BRD9 bromodomain. We therefore curated a relevant set of gene-distal putative enhancers from the ENCODE database of DNaseI hypersensitive sites (DHS; refs. 127, 128). We merged DHSs from CD14^+^ monocytes, NB4 acute leukemia cells, and HL60 acute leukemia cells, keeping only sites that did not overlap an annotated gene and were present in at least two of the datasets (Supplementary Table S3). To confirm that these DHSs accurately reflected the accessible chromatin landscape in the panel of AML cell lines, we performed Assay for Transposase Accessible Chromatin (ATAC-seq) and visualized chromatin accessibility over these loci (Supplementary Table S3; refs. 103, 129). We identified strong enrichment of ATAC-seq signal over these DHSs (Supplementary Fig. S3A; Supplementary Table S3), confirming that they accurately match open chromatin regions in AML cell lines.

**FIGURE 4:**
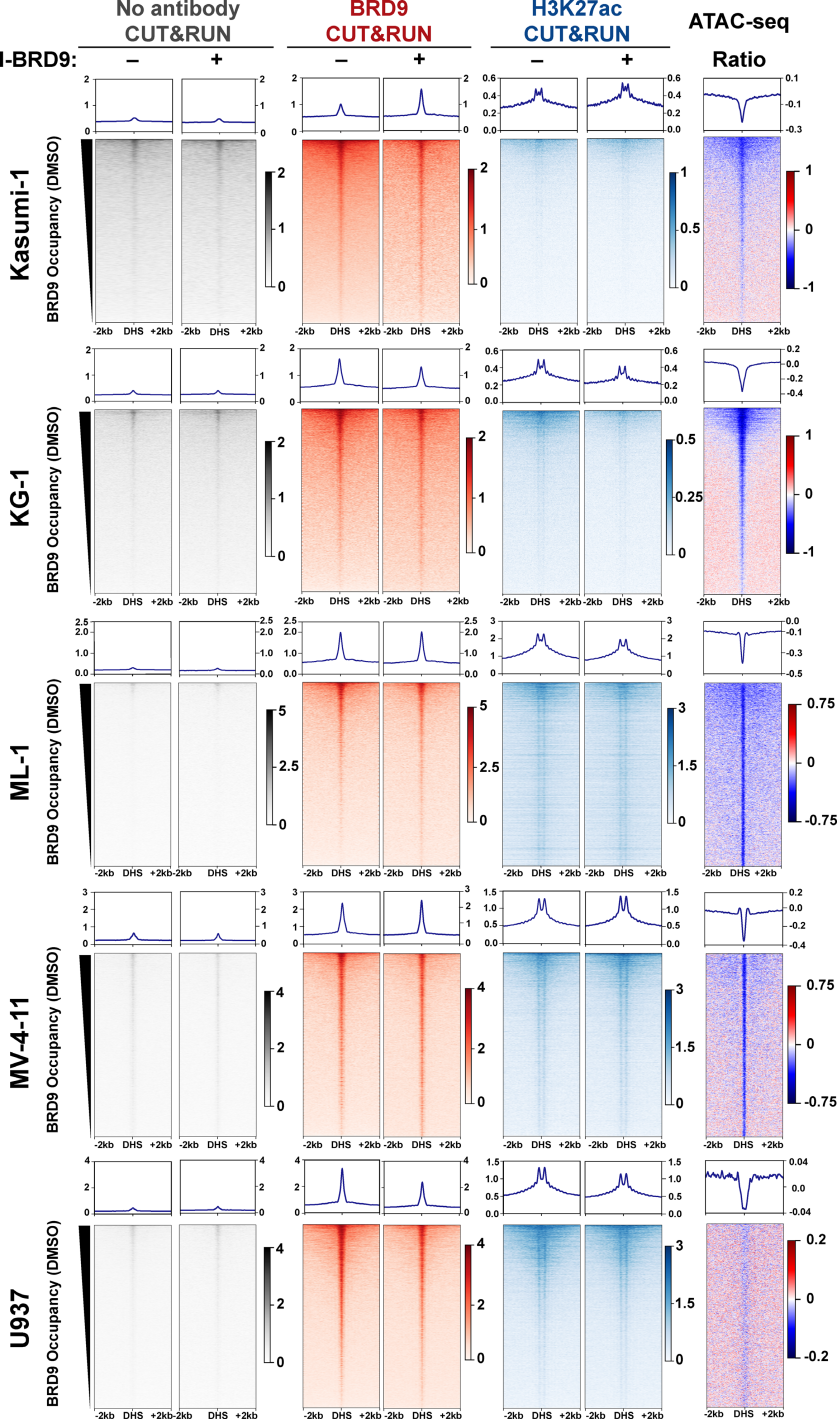
ncBAF maintains accessible chromatin at putative enhancers through BRD9’s bromodomain. From left to right, heat maps represent untargeted CUT&RUN (gray), anti-BRD9 CUT&RUN (red), and H3K27ac CUT&RUN (blue), each performed under vehicle (DMSO; “–”) or 10 µmol/L I-BRD9 (“+”) treatment. At right, plots depict the ratio of ATAC-seq signal in I-BRD9–treated samples to DMSO-treated samples. All data are sorted by BRD9 occupancy in the DMSO-treated samples for each cell line. For each experiment, *n* = 2 averaged replicates. Each dataset is plotted over gene-distal DHSs present in at least two ENCODE DHS datasets from CD14^+^ monocytes, NB4 AML cells, or HL60 AML cells (±2 kb).

To determine whether these gene-distal DHSs represent putative enhancers, we profiled H3K27ac, a mark of active enhancers and promoters, genome-wide using CUT&RUN and visualized enrichment over these gene distal DHS regions (Fig. 4; Supplementary Fig. S3A and S3B; refs. 93, 94, 96). We observed strong enrichment of H3K27ac signal at these DHSs, supporting that these locations are putative enhancers (Fig. 4; Supplementary Table S4). We next examined whether ncBAF binds these putative enhancers by performing CUT&RUN for BRD9 and visualizing enrichment over the same regions (Fig. 4; Supplementary Fig. S3; Supplementary Table S4). We observed BRD9 occupancy at these putative enhancers where H3K27ac occupancy qualitatively correlated with BRD9 occupancy (Fig. 4). Together, these data demonstrate that across a set of curated putative enhancers, we observe accessible chromatin, H3K27ac localization, and ncBAF occupancy. We next hypothesized that BRD9 bromodomain was working through one of three mechanisms: regulating localization of ncBAF, regulating H3K27ac levels, or regulating chromatin accessibility at these putative enhancers.

To test whether BRD9 bromodomain activity inhibition alters ncBAF occupancy, we performed CUT&RUN following 6 hours of I-BRD9 treatment. Surprisingly, when examined in aggregate, we detected only modest reduction in BRD9 occupancy of putative enhancers in three of five cell lines, with little change in ML-1 and an increase in BRD9 occupancy in Kasumi-1 (Fig. 4). We also observed only modest changes to H3K27ac occupancy at these locations (Fig. 4; Supplementary Table S4). To test whether BRD9 bromodomain inhibition altered chromatin accessibility at these enhancers, we performed ATAC-seq following 6 hours of I-BRD9 treatment. Inhibition resulted in reduced chromatin accessibility at enhancers in all five cell lines, suggesting a filling-in of nucleosomes at these enhancers (Supplementary Table S3). Together, these data suggest that the BRD9 bromodomain is important for the remodeling function of ncBAF, but at least partially dispensable for complex occupancy on chromatin. The magnitude of change in accessibility is strongest at the enhancers with the most BRD9 occupancy, and enrichment of H3K27ac is qualitatively correlated with both BRD9 occupancy and altered chromatin accessibility (Fig. 4). This trend is consistent with distinct roles for BRD9 in the ncBAF complex as an epigenetic reader and as a scaffolding protein essential for full complex assembly.

To determine whether the trend observed at enhancers was recapitulated at promoter elements, we analyzed the same CUT&RUN and ATAC-seq datasets over annotated TSSs (Supplementary Figs. S4 and S5; Supplementary Tables S5 and S6). Overall, we observed similar trends, where neither BRD9 nor H3K27ac occupancy were altered following I-BRD9 treatment, but chromatin accessibility was reduced. Qualitatively, we observe correlation between BRD9 occupancy and H3K27ac occupancy at promoter regions, regardless of whether BRD9 was inhibited or not but the greatest effect observed is over those locations bound by BRD9 and decorated with H3K27ac (Supplementary Fig. S5; Supplementary Tables S5 and S6). Together, these data suggest that BRD9 bromodomain activity is required for maintaining open chromatin at both enhancers and promoters but is not required for BRD9 localization to the same regions.

Finally, we examined the subset of locations at which BRD9 occupancy is directly dependent on the protein's bromodomain in each cell line. To do so, we merged peaks from both I-BRD9–treated and DMSO-treated CUT&RUN samples, then assessed differential peak occupancy using HOMER getDifferentialPeaks (130). Peaks which were significantly enriched (score 2-fold higher, *P* < 0.01) in DMSO-treated samples over I-BRD9–treated samples were called on a cell line–specific basis. While BRD9 remained bound to many loci, there was a sharp decrease in overall BRD9 occupancy in each cell line, suggesting a critical role for the BRD9 bromodomain in preserving interaction at these loci (Supplementary Fig. S6). Intriguingly, these regions contained a lower proportion of promoters than overall BRD9 CUT&RUN peaks from each cell line, while regions containing annotated noncoding RNAs were enriched in all cell lines with more than 100 bromodomain-dependent BRD9 CUT&RUN peaks (KG-1, ML-1, MV-4-11, and U937). Together, these data suggest that the ncBAF complex may require the acetyl-lysine sensing function of the BRD9 bromodomain to sustain AML cell viability. To test this hypothesis, we examined a well-studied paradigm of enhancer-mediated oncogene expression in AML: the regulation of *Myc* through its AML-specific superenhancer cluster, the Blood Enhancer Cluster (BENC).

### BRD9 Bromodomain Activity Regulates Expression of Myc Through the BENC Superenhancer

**FIGURE 5:**
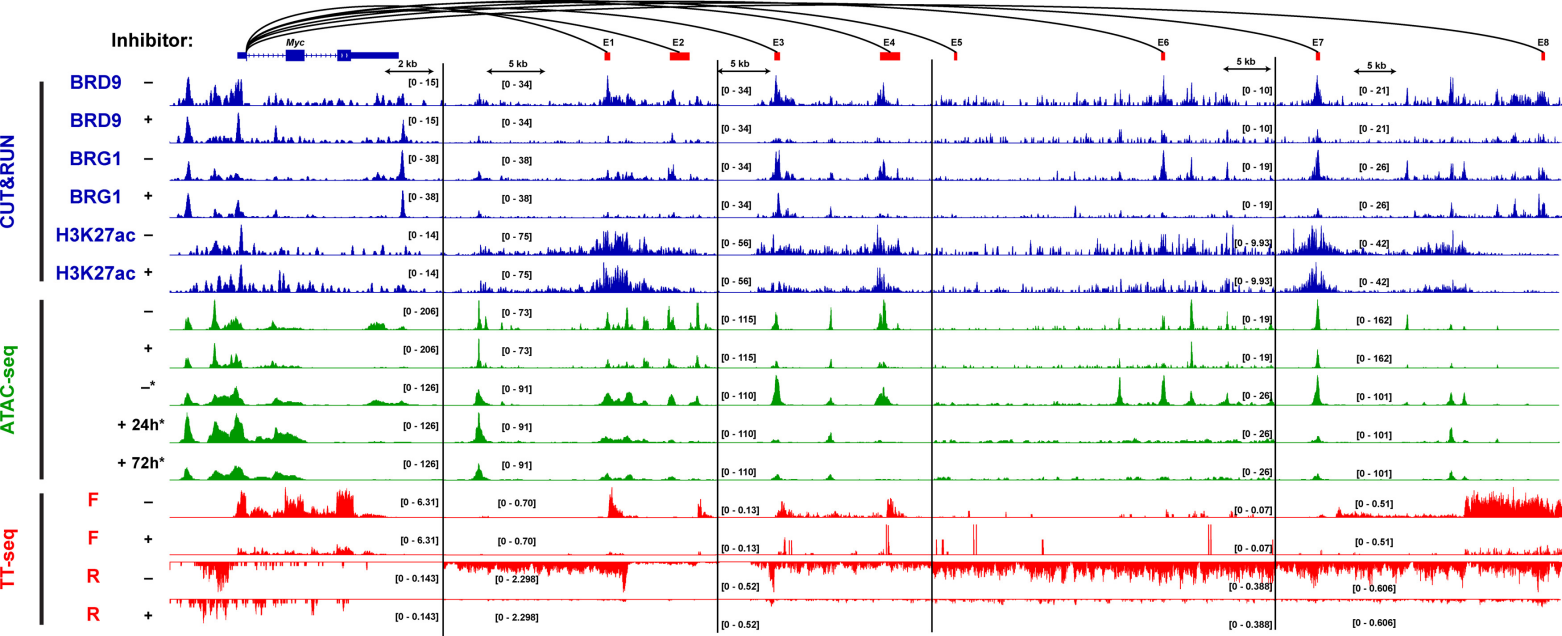
*Myc* expression in AML is dependent on BRD9-mediated enhancer interaction. Genome browser track depicting ncBAF complex occupancy, H3K27ac localization, chromatin accessibility, and nascent transcription at the *Myc* genomic locus and the AML-specific *Myc* superenhancer BENC. Changes in chromatin accessibility after 6-hour BRD9 inhibition mirror those caused by prolonged inhibition of BAF complex ATPases (8). *n* = 2 replicates per track (averaged). ATAC-seq tracks marked with an asterisk (*) are from GSE190722. All experiments shown were performed in the MV-4-11 cell line and are consistent with other cell lines. See Supplementary Fig. S7 for ML-1 cell line data.

*Myc* is a well-studied oncogene that is activated in many cancers, with roles in cancer progression, tumor growth via “oncogene addiction,” and immune system evasion (131). Despite having low mutational rates, *Myc* is often overexpressed in AML—even in as many as 90% of AML blasts (18, 131, 132). Intriguingly, PU.1 and MYC regulate response to *Kit* and *Lsd1* inhibitors (132), implying a possible mechanism through which ncBAF regulates *Kit*, *Bcl2*, *Btk1*, and other druggable targets. Given the established relationships between PU.1, MYC, and BRD9 (4, 8, 132), and our data indicating a role for BRD9 in regulating *Myc* and *Kit* gene expression (Fig. 2; Supplementary Fig. S1), we attempted to define a more pointed paradigm of BRD9-mediated oncogene regulation. To understand how BRD9 may contribute to *Myc* expression, we examined our CUT&RUN, ATAC-seq, and TT-seq data at the *Myc* gene and its associated distal superenhancer common to AML, BENC. We identified strong enrichment of BAF complex subunits at individual BENC enhancers in vehicle control CUT&RUN samples with reduced BRG1 and BRD9 occupancy after I-BRD9 treatment, suggesting that acetyl-lysine recognition by BRD9 is necessary to recruit or retain the ncBAF complex at these enhancers (Fig. 5; Supplementary Fig. S7, blue tracks). This is in stark contrast with genome-wide analyses, which showed no change in BRD9 occupancy at a majority of putative AML enhancers (Fig. 4).

Transcription of the *Myc* gene was reduced after BRD9 inhibition, in line with prior results in murine and human AML cell lines (refs. 4, 5; Fig. 5; Supplementary Fig. S7, red tracks); however, BAF subunit binding was unchanged at the *Myc* promoter, supporting a mechanism of gene-distal regulation by ncBAF. We visualized our ATAC-seq data alongside public ATAC-seq data from the same cell line (MV-4-11) treated with the BRM014 inhibitor which inhibits both BRM and BRG1 ATPase activity in all BAF complexes (8). Accessibility of the *Myc* promoter was unchanged by 24-hour treatment with BRM014 (Fig. 5, green tracks), and modestly reduced by 6-hour I-BRD9 treatment (Fig. 5; Supplementary Fig. S7, green tracks). BENC enhancers, however, displayed reduced accessibility after treatment with either BRM014 or I-BRD9 when compared with vehicle controls (Fig. 5; Supplementary Fig. S7). Intriguingly, we also identified a reduction in transcription of eRNA from the BENC enhancers after I-BRD9 treatment (Fig. 5; Supplementary Fig. S7). Collectively, these data demonstrate extensive dysregulation of the AML-specific BENC *Myc* enhancer cluster, providing a mechanism through which *Myc* transcription is reduced by BRD9 inhibition. Because of the AML-specific requirement for the BRD9 bromodomain and the precise regulation of *Myc* through its AML-specific enhancers—but not all myeloid enhancers—we hypothesize that BRD9 is responsible for maintenance of an AML-specific subset of genes—including *Myc*—through gene-distal regulation.

### BRD9 Bromodomain Activity Facilitates AP-1, ETS, and GATA Family TF Binding

**FIGURE 6:**
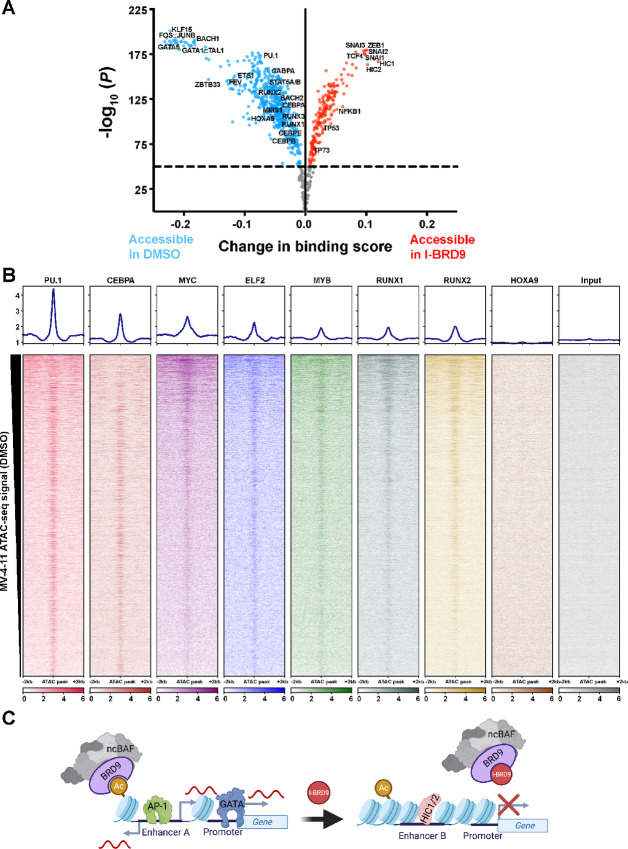
ncBAF maintains accessible chromatin at gene-distal regulatory elements bound by hematopoietic TFs. **A,** Volcano plot depicting selected motifs with altered ATAC-seq footprints after BRD9 inhibition. All cell lines were merged for this analysis (*n* = 10 per condition). **B,** Analysis of published AML core circuit factor ChIP-seq data from PRJNA751732 (102), visualized over bromodomain-dependent ATAC-seq peaks. All experiments were performed in the MV-4-11 cell line. Average values of two to three independent replicates are shown in each heat map. **C,** Proposed model of ncBAF-mediated hematopoietic TF regulation.

To better characterize the link between the AML-specific role of BRD9 (myeloid immaturity and AML cell growth) and the genomic effects of BRD9 inhibition (reduced chromatin accessibility at *cis*-regulatory regions and dysregulated transcription), we performed TF accessibility footprinting analysis using our ATAC-seq data through TOBIAS (105). TOBIAS integrates genomic information with differential chromatin accessibility to predict TF binding genome-wide at validated DNA sequence motifs (105). We analyzed differential accessibility at TF DNA sequence motifs included in the JASPAR2022 vertebrate core collection (106). Hematopoietic TFs associated with immature myeloid cells displayed significantly decreased accessibility, including the GATA, ETS, CEBP, RUNX, and BACH families (Fig. 6A). Intriguingly, regions with increased accessibility upon I-BRD9 treatment harbored motifs associated with tumor suppressor genes, including TP53, HIC1/HIC2, and NFKB1, and SNAIL family TFs (Fig. 6A). Together, these analyses indicate a role for BRD9 in preventing tumor suppressor proteins from binding to chromatin, while simultaneously regulating hematopoietic differentiation state.

To more precisely understand the connection between AML and regulation by the ncBAF complex, we downloaded publicly available ChIP-seq data from the MV-4-11 cell line, targeting factors involved in an AML core regulatory circuit (SRA: PRJNA751732; ref. 102). We visualized these data over regions that we identified as accessible peaks in DMSO-treated but not I-BRD9–treated MV-4-11 cells and observed consistent binding that qualitatively correlates with the level of accessibility (Fig. 6B). We interpret this trend as evidence of BRD9-dependent regulation of the core AML circuitry, with strong overlap between BRD9 bromodomain-dependent accessible regions of chromatin and PU.1, CEBPa, and MYC, among others. It is possible, however, that maintenance of accessibility at these regions is not necessary for these factors, as most have been described or theorized to be pioneer factors. Alternatively, maintenance of open chromatin at these regions may be dependent on ncBAF-stimulated transcription of the indicated factors, rather than by direct ncBAF activity at the indicated loci. Indeed, BRD9 inhibition significantly reduced transcription of all but *Sp1, Elf2,* and *HOXA9* in MV-4-11 (Supplementary Table S2), strengthening the possibility of indirect regulation by ncBAF.

## Discussion

In this study, we identified genic and gene-distal regulation of chromatin accessibility by the ncBAF complex in five AML cell lines, directed through the bromodomain of the complex subunit BRD9. We propose a model wherein BRD9 facilitates binding of hematopoietic TFs to maintain AML cells in an incompletely differentiated state (Fig. 6C). In particular, inhibition of BRD9 bromodomain acetyl-lysine recognition disrupted hematopoietic TF footprints, reducing accessibility at sequence motifs recognized by GATA, ETS, and AP-1 family members, while sequence motifs recognized by SNAIL-, HIC-, and TP53-related TFs became more accessible when BRD9 was inhibited (Fig. 6A). Disruption of BRD9 bromodomain at enhancers led to drastically altered transcription of the enhancers’ genic targets—such as *Myc*—and was accompanied by a loss of chromatin accessibility at both putative enhancers and promoters, though BRD9 binding was only moderately disrupted globally (Figs. 4 and 5). In support of a specific role for BRD9 in AML maintenance, treatment with I-BRD9 alone is selectively lethal to five distinct AML cell lines but not to HEK293T cells (Fig. 1). This specific dependency on BRD9 for AML cell growth may be perpetuated through the CCR2-CCL2 tumor proliferation axis (Fig. 2C and D).

While large-scale screens have identified BAF complex subunits as leukemia-specific dependencies (64, 133) and BAF complex ATPase inhibitors have shown ability to selectively inhibiting leukemia cell growth (8), the BAF complex has essential roles in normal hematopoiesis (51, 61, 134), complicating the process of targeting BAF ATPases for treating AML. To exploit BAF's essential roles in AML cells for cancer therapy, a more precise and specific target must be identified. The BAF subunit BRD9 is overexpressed in AML cells, regulates only a subset of BAF complex functions, and is necessary for AML cell viability; as such, BRD9 is an appealing target for drug therapy. Competitive inhibition of the BRD9 bromodomain allows precise disruption of ncBAF complex function, avoiding the off-target effects of BAF ATPase disruption and knockdown-related issues with complex assembly. A potential scaffolding role for BRD9 in ncBAF assembly may explain the more drastic dissociation from chromatin after BRD9 knockdown than BRD9 inhibition (66).

Transcriptomic analysis of BRD9 inhibition suggests roles for ncBAF in cell-cycle progression, myeloid differentiation, and suppression of tumor suppressor genes. Given the extensive overlap between BRD9 inhibition and FDA-approved AML chemotherapy targets, the ability of I-BRD9 to specifically inhibit AML cell growth, and the release from impaired differentiation prompted by I-BRD9 treatment, BRD9 bromodomain inhibition may provide a tractable chemotherapeutic opportunity for AML treatment. Because BRD9 maintains expression of the targets of approved chemotherapy drugs—including venetoclax, ibrutinib, imatinib, and gemutuzab (Fig. 2E; Supplementary Fig. S1G and S1H)—the protein may be most usefully targeted in combination with other chemotherapy drugs, perhaps as a treatment avenue for patients that have acquired resistance to other treatments.

## Supplementary Material

Table S1 Table S1. Variants identified in selected BAF complex subunit genes by whole-gene sequencing of a targeted panel, including Arid1a, Arid1b, Bcl7a, Bcl7b, Bcl11a, Bcl11b, Brd9, Smarca2, and Smarca4.

Table S2 Table S2. A complete analysis of TT-seq data generated in this study.

Table S3 Table S3. Genomic coordinates and normalized signal over curated myeloid gene-distal DHSs for all ATAC-seq experiments.

Table S4 Table S4. Genomic coordinates and normalized signal over curated myeloid gene-distal DHSs for all CUT&RUN experiments.

Table S5 Table S5. Normalized signal over Gencode V38 promoters for all ATAC-seq experiments.

Table S6 Table S6. Normalized signal over Gencode V38 promoters for all CUT&RUN experiments.

Supplementary Figure 1 Quality-control and reproducibility information for TT-seq experiments

Supplementary Figure 2 The BRD9 bromodomain regulates transcription of hematopoiesis-related genes

Supplementary Figure 3 Analysis of chromatin environment at gene-distal DHSs

Supplementary Figure 4 BRD9 inhibition leads to reduced chromatin accessibility at mRNA TSSs

Supplementary Figure 5 ncBAF maintains accessible chromatin at TSSs through BRD9’s bromodomain

Supplementary Figure 6 The BRD9 bromodomain is responsible for maintaining BRD9 occupancy on chromatin at a cell line-specific subset of BRD9-bound regions

Supplementary Figure 7 BRD9 bromodomain activity regulates accessibility and transcription from BENC enhancers in the ML-1 cell line
