# Supplementary material for: The ncBAF Complex Regulates Transcription in AML Through H3K27ac Sensing by BRD9
Source: Cancer Res Commun. 2024 Jan 30;4(1):237–52. doi: 10.1158/2767-9764.CRC-23-0382 (PMC10831031; doi:10.1158/2767-9764.CRC-23-0382)
Supplement: Supplementary Figure 1 — Quality-control and reproducibility information for TT-seq experiments [file crc-23-0382-s07.pdf]

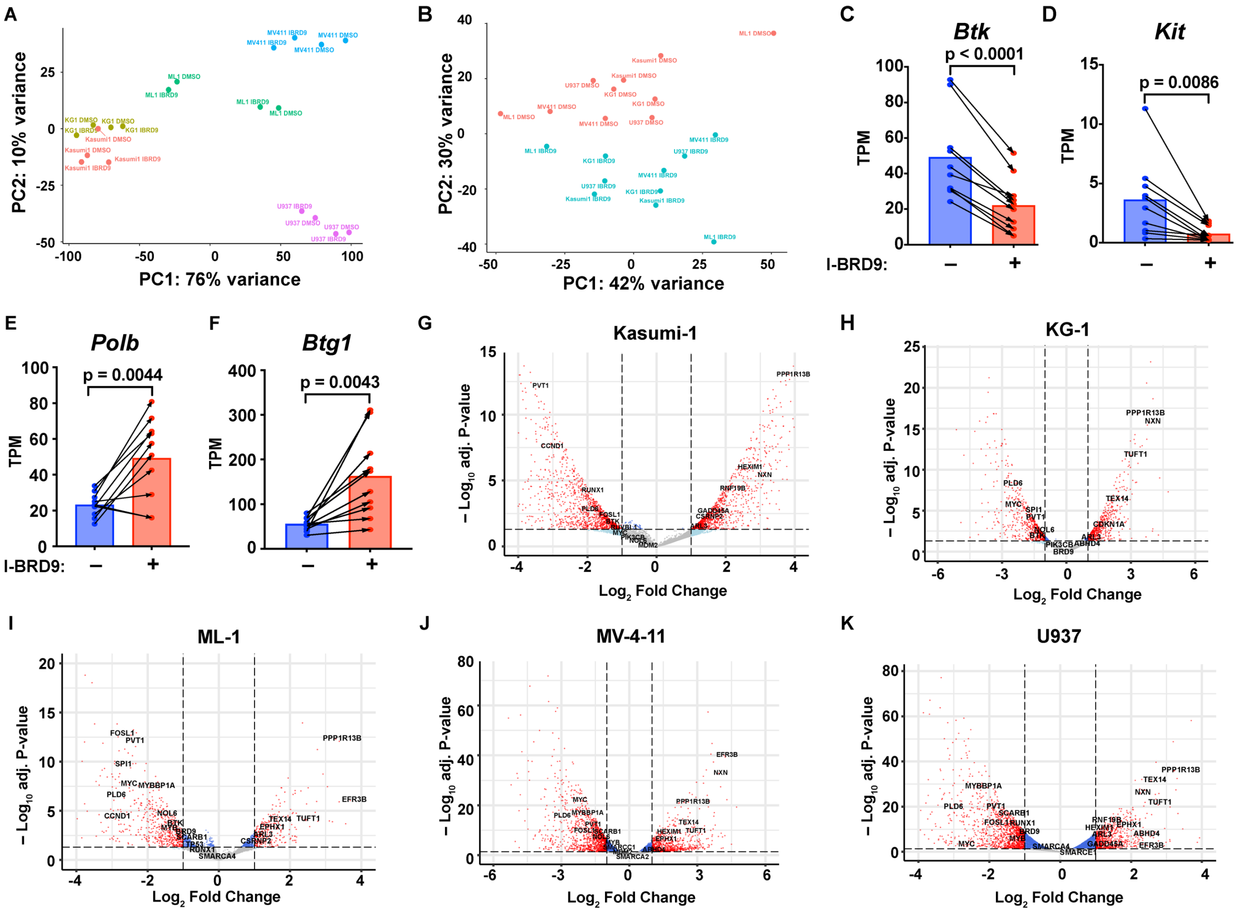

Figure S1. Quality-control and reproducibility information for TT-seq experiments. A-B. Principal component analysis depicting relationships between TT-seq replicates. No corrections were applied to the plot in panel A, while the plot in panel B was batch-corrected for cell line but not treatment-specific effects using limma. **C-F.** Transcripts-per-million (TPM) values of targets of FDA-approved chemotherapies for leukemia (C,D) and tumor suppressor genes (E,F). Arrows link the same replicate between conditions. n = 2 replicates per drug condition and cell line, for a total of 20 experiments. Significance was assessed through paired two-tailed t-tests. **G-K.** Volcano plots depicting differentially transcribed genes for each individual cell line as in Figure 2. Fold changes are shown as effect after 10 μM I-BRD9 treatment in I-BRD9, relative to vehicle (DMSO). Significance cutoffs of adj. $p < 0.05$ and $|\log_2$ fold change$| > 1$ are indicated by dashed lines. n = 2 replicates per drug condition.
