# Supplementary material for: The ncBAF Complex Regulates Transcription in AML Through H3K27ac Sensing by BRD9
Source: Cancer Res Commun. 2024 Jan 30;4(1):237–52. doi: 10.1158/2767-9764.CRC-23-0382 (PMC10831031; doi:10.1158/2767-9764.CRC-23-0382)
Supplement: Supplementary Figure 2 — The BRD9 bromodomain regulates transcription of hematopoiesis-related genes [file crc-23-0382-s08.pdf]

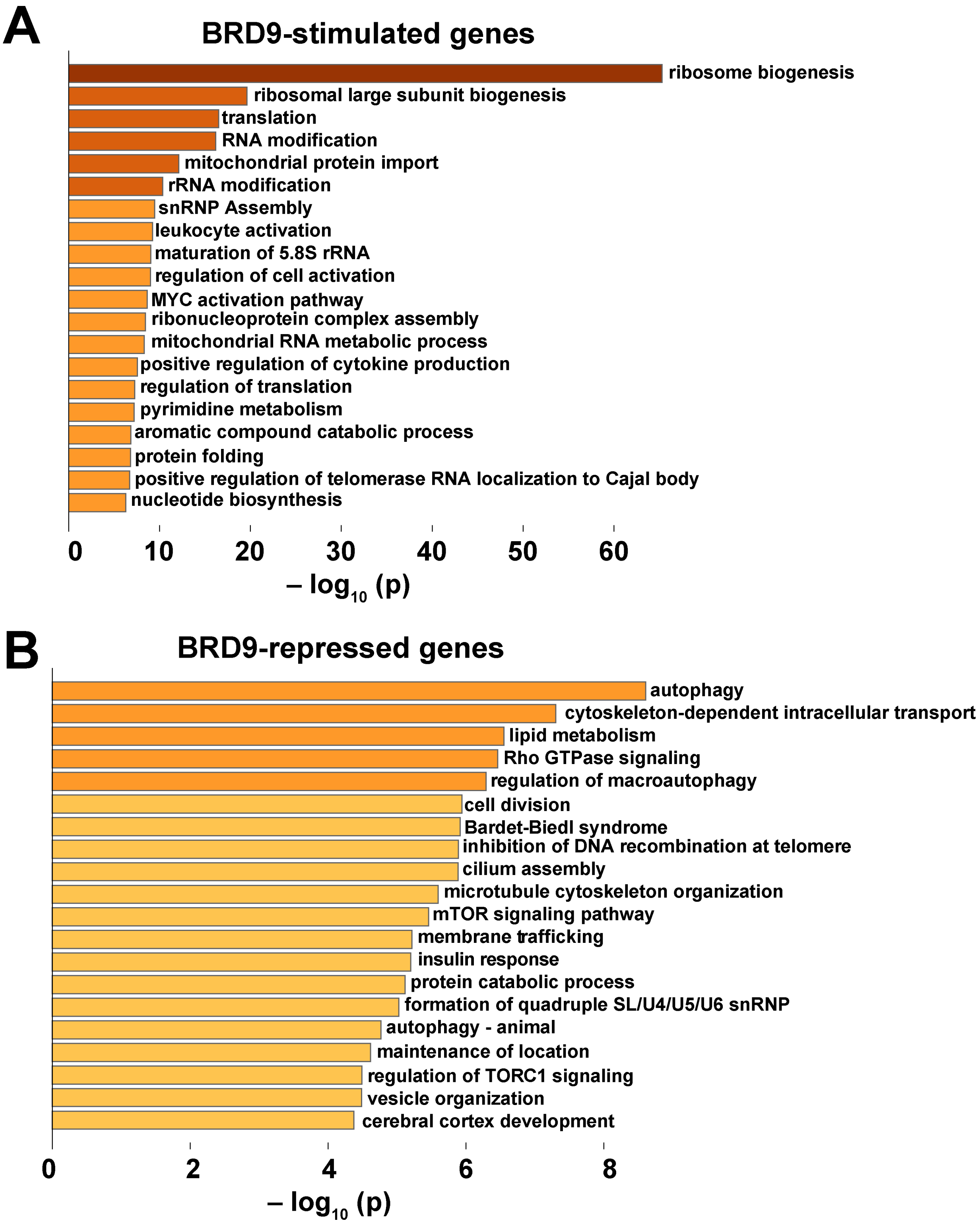

Figure S2. The BRD9 bromodomain regulates transcription of hematopoiesis-related genes. A-B. Gene ontology analysis for genes with reduced (A) or increased (B) transcription after BRD9 inhibition. Gene lists were identified from DESeq2 results (adj. $p < 0.05$) and filtered against all expressed genes in the composite AML cell line analysis (baseMean > 1). $n = 20$ total replicates, 2 per cell line.
