# Supplementary material for: The ncBAF Complex Regulates Transcription in AML Through H3K27ac Sensing by BRD9
Source: Cancer Res Commun. 2024 Jan 30;4(1):237–52. doi: 10.1158/2767-9764.CRC-23-0382 (PMC10831031; doi:10.1158/2767-9764.CRC-23-0382)
Supplement: Supplementary Figure 3 — Analysis of chromatin environment at gene-distal DHSs [file crc-23-0382-s09.pdf]

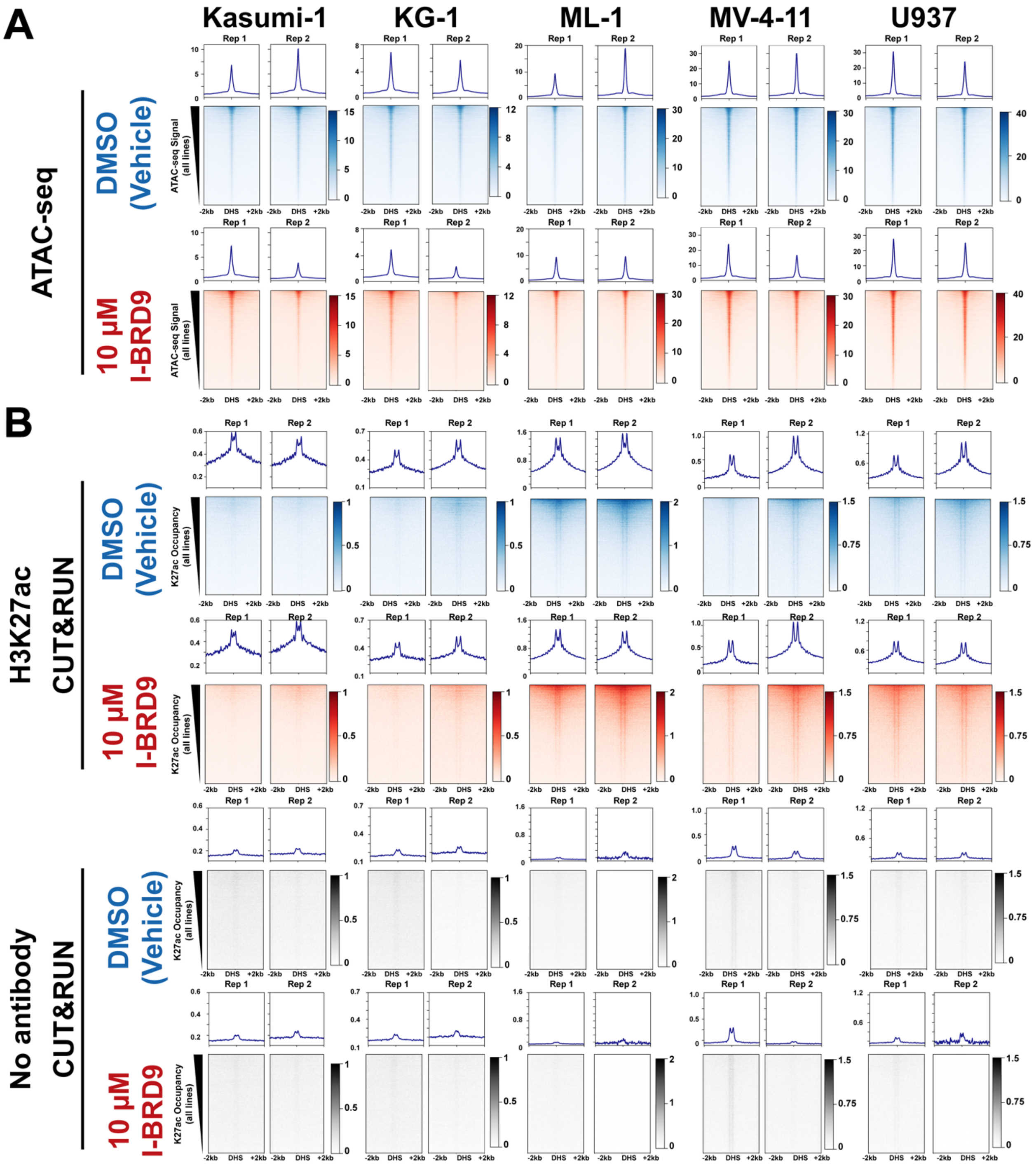

Figure S3. Analysis of chromatin environment at gene-distal DHSs. A. Individual ATAC-seq replicates, plotted over gene-distal DHSs sourced from CD14+ monocytes, NB4 leukemia cells, and HL60 leukemia cells (ENCODE). DHSs were kept only if they did not overlap a gene at any point and were present in 2/3 datasets. Data are sorted by ATAC-seq signal in all samples. Cells were treated with either 10 µM I-BRD9 or 1% DMSO (vehicle) for six hours prior to experiment. **B.** (TOP) Individual H3K27ac CUT&RUN replicates, sorted by H3K27ac occupancy across all

samples. (BOTTOM) Individual replicates of untargeted CUT&RUN (no primary antibody added), sorted by H3K27ac occupancy across all samples.
