# Supplementary material for: The ncBAF Complex Regulates Transcription in AML Through H3K27ac Sensing by BRD9
Source: Cancer Res Commun. 2024 Jan 30;4(1):237–52. doi: 10.1158/2767-9764.CRC-23-0382 (PMC10831031; doi:10.1158/2767-9764.CRC-23-0382)
Supplement: Supplementary Figure 4 — BRD9 inhibition leads to reduced chromatin accessibility at mRNA TSSs [file crc-23-0382-s10.pdf]

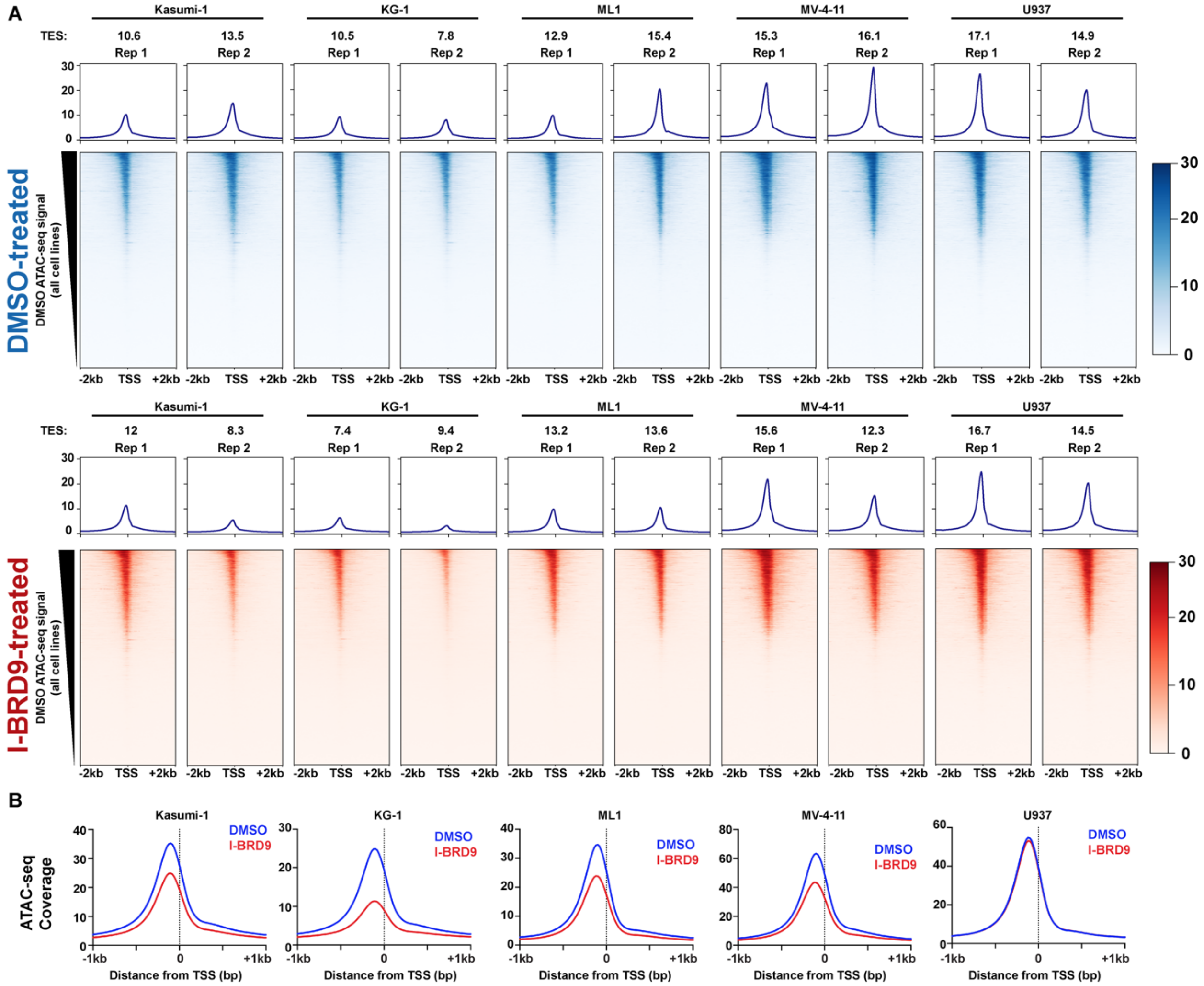

Figure S4. BRD9 inhibition leads to reduced chromatin accessibility at mRNA TSSs. A. ATAC-seq signal after vehicle (DMSO, blue, top) or 10 µM I-BRD9 (red, bottom) treatment, visualized at Gencode V38 annotated transcription start sites (TSSs), ±2kb. n = 2 replicates per vehicle treatment, shown individually. **B.** Replicate-merged ATAC-seq signal at RefSeq Select annotated mRNA TSSs, ±1kb. In 4/5 cell lines, BRD9 inhibition led to a strong reduction in chromatin accessibility at promoters, while effects were consistent but muted in U937. n = 2 averaged replicates per drug treatment.
