# Supplementary material for: The ncBAF Complex Regulates Transcription in AML Through H3K27ac Sensing by BRD9
Source: Cancer Res Commun. 2024 Jan 30;4(1):237–52. doi: 10.1158/2767-9764.CRC-23-0382 (PMC10831031; doi:10.1158/2767-9764.CRC-23-0382)
Supplement: Supplementary Figure 5 — ncBAF maintains accessible chromatin at TSSs through BRD9’s bromodomain [file crc-23-0382-s11.pdf]

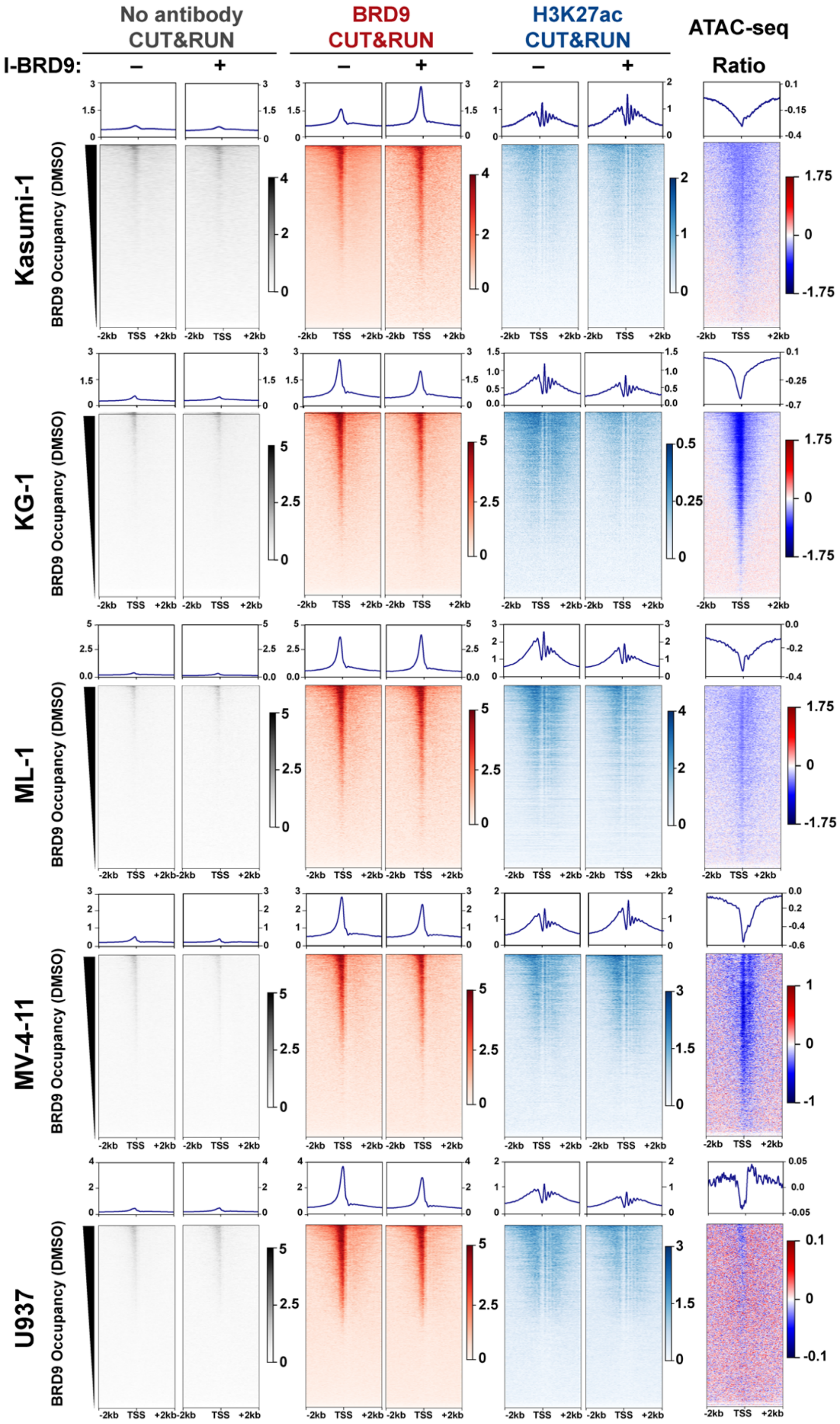

Figure S5. ncBAF maintains accessible chromatin at TSSs through BRD9's bromodomain. From left to right, plots represent untargeted CUT&RUN (gray), BRD9

CUT&RUN (red), and H3K27ac CUT&RUN (blue), each performed under vehicle (DMSO) or 10 μM I-BRD9 treatment. At right, plots depict the ratio of ATAC-seq signal in I-BRD9-treated samples relative to DMSO-treated samples. For each experiment, n = 2 averaged replicates. Each dataset is plotted over RefSeq select mRNA annotated TSSs, ±2kb. Data are sorted by BRD9 occupancy in the DMSO-treated samples for each cell line.
