# Supplementary material for: The ncBAF Complex Regulates Transcription in AML Through H3K27ac Sensing by BRD9
Source: Cancer Res Commun. 2024 Jan 30;4(1):237–52. doi: 10.1158/2767-9764.CRC-23-0382 (PMC10831031; doi:10.1158/2767-9764.CRC-23-0382)
Supplement: Supplementary Figure 6 — The BRD9 bromodomain is responsible for maintaining BRD9 occupancy on chromatin at a cell line-specific subset of BRD9-bound regions [file crc-23-0382-s12.pdf]

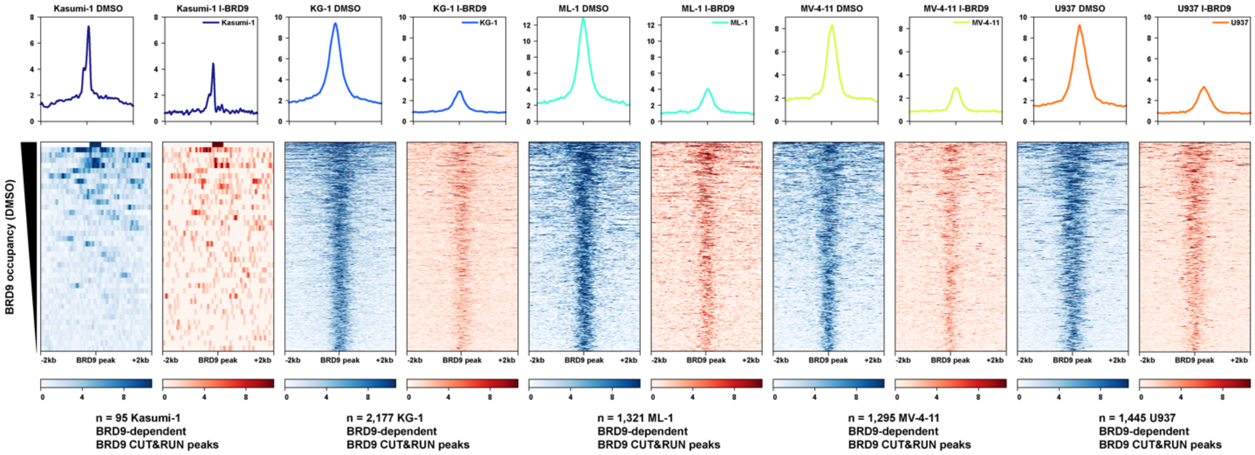


**Figure S6. The BRD9 bromodomain is responsible for maintaining BRD9 occupancy on chromatin at a cell line-specific subset of BRD9-bound regions.** Peaks that were present in DMSO- but not I-BRD9-treated BRD9 CUT&RUN were isolated from each cell line and sorted by BRD9 occupancy in DMSO-treated samples. BRD9 CUT&RUN data were visualized in each cell line over the cell line-specific BRD9-dependent CUT&RUN peaks. n = 2 BRD9 CUT&RUN replicates per track (averaged).
