# Supplementary material for: The ncBAF Complex Regulates Transcription in AML Through H3K27ac Sensing by BRD9
Source: Cancer Res Commun. 2024 Jan 30;4(1):237–52. doi: 10.1158/2767-9764.CRC-23-0382 (PMC10831031; doi:10.1158/2767-9764.CRC-23-0382)
Supplement: Supplementary Figure 7 — BRD9 bromodomain activity regulates accessibility and transcription from BENC enhancers in the ML-1 cell line [file crc-23-0382-s13.pdf]

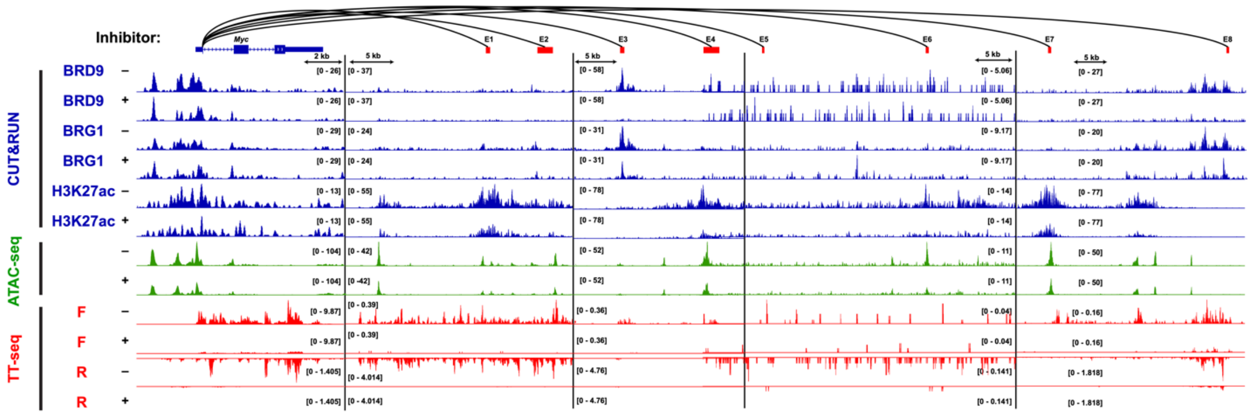

Figure S7. BRD9 bromodomain activity regulates accessibility and transcription from BENC enhancers in the ML-1 cell line. Genome browser track depicting ncBAF complex occupancy (BRD9 or BRG1 CUT&RUN), H3K27ac (CUT&RUN), chromatin accessibility (ATAC-seq), and nascent transcription (TT-seq, forward and reverse strands shown independently, labeled as F or R, respectively) at the *Myc* genomic locus and the AML-specific *Myc* superenhancer BENC. n = 2 replicates per track (averaged). All experiments shown were performed in the ML-1 cell line and are consistent with other cell lines.
